# Microglia Negatively Regulate the Proliferation and Neuronal Differentiation of Neural Stem/Progenitor Cells Isolated from Poststroke Mouse Brains

**DOI:** 10.3390/cells12162040

**Published:** 2023-08-10

**Authors:** Yoshinobu Hirano, Takayuki Nakagomi, Akiko Nakano-Doi, Shuji Kubo, Yusuke Minato, Toshinori Sawano, Masafumi Sakagami, Kenzo Tsuzuki

**Affiliations:** 1 Department of Otorhinolaryngology—Head & Neck Surgery, Hyogo Medical University, 1-1 Mukogawa, Nishinomiya 663-8501, Japan; yo-hirano@hyo-med.ac.jp (Y.H.); msakaga@hyo-med.ac.jp (M.S.); kenzo@hyo-med.ac.jp (K.T.); 2 Institute for Advanced Medical Sciences, Hyogo Medical University, 1-1 Mukogawacho, Nishinomiya 663-8501, Japan; nakano@hyo-med.ac.jp (A.N.-D.); s-kubo@hyo-med.ac.jp (S.K.); 3 Department of Therapeutic Progress in Brain Diseases, Hyogo Medical University, 1-1 Mukogawacho, Nishinomiya 663-8501, Japan; 4 Department of Anatomy and Cell Biology, Hyogo Medical University, 1-1 Mukogawacho, Nishinomiya 663-8501, Japan; yu-minato@hyo-med.ac.jp; 5 Department of Biomedical Sciences, Ritsumeikan University, 1-1-1 Nojihigashi, Kusatsu 525-8577, Japan; t-sawano@fc.ritsumei.ac.jp

**Keywords:** neural stem/progenitor cells, microglial cells/macrophages, ischemic stroke, neural regeneration

## Abstract

We previously demonstrated that neural stem/progenitor cells (NSPCs) were induced within and around the ischemic areas in a mouse model of ischemic stroke. These injury/ischemia-induced NSPCs (iNSPCs) differentiated to electrophysiologically functional neurons in vitro, indicating the presence of a self-repair system following injury. However, during the healing process after stroke, ischemic areas were gradually occupied by inflammatory cells, mainly microglial cells/macrophages (MGs/MΦs), and neurogenesis rarely occurred within and around the ischemic areas. Therefore, to achieve neural regeneration by utilizing endogenous iNSPCs, regulation of MGs/MΦs after an ischemic stroke might be necessary. To test this hypothesis, we used iNSPCs isolated from the ischemic areas after a stroke in our mouse model to investigate the role of MGs/MΦs in iNSPC regulation. In coculture experiments, we show that the presence of MGs/MΦs significantly reduces not only the proliferation but also the differentiation of iNSPCs toward neuronal cells, thereby preventing neurogenesis. These effects, however, are mitigated by MG/MΦ depletion using clodronate encapsulated in liposomes. Additionally, gene ontology analysis reveals that proliferation and neuronal differentiation are negatively regulated in iNSPCs cocultured with MGs/MΦs. These results indicate that MGs/MΦs negatively impact neurogenesis via iNSPCs, suggesting that the regulation of MGs/MΦs is essential to achieve iNSPC-based neural regeneration following an ischemic stroke.

## 1. Introduction

The brain comprises various cells, including those of neural lineage (e.g., neurons, astrocytes, and oligodendrocytes) and vascular lineage (e.g., endothelial cells and pericytes). Neural lineage cells are particularly vulnerable to ischemia/hypoxia and rapidly undergo cell death after an ischemic stroke [1]. However, evidence increasingly shows that neural stem/progenitor cells (NSPCs), which can differentiate into electrophysiologically functional neurons, astrocytes, and oligodendrocytes, are induced within and around ischemic areas following stroke [2,3], implicating the capability of endogenous injury/ischemia-induced NSPCs (iNSPCs) to restore damaged neural cells after an ischemic stroke.

In addition to iNSPC induction, inflammatory cells such as microglial cells (MGs) and macrophages (MΦs), which play an important role in tissue repair following various types of brain injury [4,5], emerge within and around the affected sites. Although their precise role in pathologic processes underlying ischemic injury remains unclear, MGs/MΦs gradually accumulate within and around the injured areas and finally occupy these regions after an ischemic stroke [6]. In contrast, we previously demonstrated that the number of iNSPCs in areas affected by an ischemic stroke declined at later time points following injury [2,3]. Although the roles of MGs/MΦs in the function of endogenous NSPCs remain unclear, previous studies examining embryonic and fetal stages of brain development showed that MGs/MΦs inhibited the number of NSPCs to suppress excessive neurogenesis through several mechanisms such as phagocytosis, inhibition of cell proliferation, and promotion of cell death [7,8,9]. These results lead us to hypothesize that MGs/MΦs regulate the number of iNSPCs and neurogenesis under pathological conditions such as ischemic stroke.

In the present study, we utilized iNSPCs isolated from the site of injury in a mouse model of ischemic stroke to investigate whether brain-derived MGs/MΦs impacted the fate of iNSPCs in coculture experiments. We found that the presence of MGs/MΦs suppressed iNSPC proliferation and reduced neuronal differentiation of iNSPCs, which were not observed in iNSPCs coincubated with other cell types including astrocytes (ACs), endothelial cells (ECs), and pericytes (PCs). Additionally, MG/MΦ depletion using clodronate liposomes attenuated the MG/MΦ-mediated effects on iNSPCs. These results show that the regulation of MGs/MΦs can be a potential strategy to accomplish iNSPC-based neural regeneration following an ischemic stroke.

## 2. Materials and Methods

### 2.1. Induction of Ischemic Stroke

Experimental procedures were approved by the Animal Care Committee of Hyogo Medical University (approval no: 20-030; 23-006AG). Adult (6–10 weeks-old) male CB-17 wild-type mice (CB-17/Icr-+/+Jcl; Clea Japan, Tokyo, Japan) were used to establish the mouse model of permanent focal cerebral ischemia by ligating and interrupting the distal portion of the left middle cerebral artery (MCA) as previously described [1,2,3,10]. Briefly, in animals under isoflurane anesthesia, a burr hole was made in the skull using a drill (H021 Minimo; Minitor Tokyo, Japan), dura mater was opened, MCA occlusion (MCAO) was created by electrocoagulation, and the distal portion of the left MCA was disconnected.

### 2.2. Preparation of Brain Samples

At the time of tissue harvest, mice were intraperitoneally administered a mixture containing medetomidine (0.3 mg/kg), midazolam (4 mg/kg), and butorphanol (5 mg/kg) [2,6], followed by transcardial perfusion with 4% paraformaldehyde [1,3,10]. The brains were removed and placed in 4% paraformaldehyde for 24 h, followed by incubation in 30% sucrose for cryoprotection at −80 °C. Coronal sections cut using a cryostat were used for immunohistochemical analyses.

### 2.3. Immunohistochemistry

Using previously described immunohistochemistry protocols [1,2,3,10], 20-μm-thick coronal sections were incubated with primary antibodies against nestin (1:200, mouse; Millipore, Billerica, MA, USA) and ionized calcium-binding adapter molecule 1 (Iba-1; 1:500, rabbit; Abcam, Cambridge, UK), followed by incubated with Alexa Fluor 488- or Alexa Fluor 555-conjugated secondary antibodies (1:500; Molecular Probes, Eugene, OR, USA). Nuclei were counterstained with 4′,6-diamidino-2-phenylindole (DAPI; 1:500; Kirkegaard & Perry Laboratories, Gaithersburg, MD, USA). Images were captured using a confocal laser microscope (LSM780; Carl Zeiss, Oberkochen, Germany).

Positive areas for nestin and Iba-1 were evaluated in ischemic and peri-ischemic areas in coronal brain sections obtained from the same region across all animals. A total of 27 data points (3 areas/section and 3 sections/brain, 3 animals/each day) using ImageJ, as previously described [1,6]. Ischemic and peri-ischemic areas were defined as regions within the borders of stroke-affected areas and regions within a diameter of 100 µm around the stroke-affected areas, respectively, as described [1,6].

### 2.4. Cell Culture

Regionally-derived endogenous iNSPCs were isolated from ischemic areas of mice after MCAO and maintained in Dulbecco’s modified Eagle’s medium/F12 (Thermo Fisher Scientific, Waltham, MA, USA) containing 20 ng/mL basic fibroblast growth factor (bFGF; Peprotech, Rocky Hill, NJ, USA), 20 ng/mL epidermal growth factor (EGF; Peprotech), 1% N2 (Thermo Fisher Scientific), and 2% fetal bovine serum (FBS), as previously described [11]. Commercially available mouse brain MGs/MΦs (#SCC134; EMD Millipore, Temecula, CA, USA), ACs (#M1800; ScienCell Research Laboratories, Carlsbad, CA, USA), PCs (#M1200; ScienCell Research Laboratories), and ECs (CRL-2299; ATCC, Manassas, VA, USA) were maintained in appropriate media in accordance with the manufacturer’s protocols, as described [6]. In some experiments, iNSPCs transfected with GFP-expressing lentivirus vectors and MGs/MΦs, ACs, ECs, and PCs transfected with mCherry-expressing lentivirus vectors were used [6,10,12].

iNSPCs were cocultured with MGs/MΦs to investigate the effect of MGs/MΦs on iNSPC proliferation. Briefly, iNSPCs alone or in coculture with MGs/MΦs were plated on poly-D-lysine-coated dishes and incubated in Dulbecco’s modified Eagle’s medium/F12 containing bFGF, EGF, N2, and 2% FBS. The monocultures and cocultures were fixed and immunostained with antibodies against nestin (1:200, mouse; Millipore), Sox2 (1:100, rabbit; Abcam), Ki67 (1:50, mouse; BD Pharmingen, San Diego, CA, USA), and mCherry (1:500, rabbit or 1:500, chicken; Abcam), followed by incubation with Alexa Fluor 488- or Alexa Fluor 555-conjugated secondary antibodies (1:500; Molecular Probes). Nuclei were counterstained with DAPI (1:500), and fluorescence images were captured using a laser scanning microscope (LSM780). The number of nestin^+^ iNSPCs (nestin^+^/mCherry^−^ cells) or Sox2^+^ iNSPCs (Sox2^+^/mCherry^−^ cells) was compared between iNSPC monocultures (control) and iNSPCs cocultured with mCherry^+^ MGs/MΦs using 12 data points (4 areas/sample, 3 samples/marker), as described [13]. Additionally, the ratio of Ki67^+^ iNSPCs (Ki67^+^/mCherry^−^ cells) to all iNSPCs (DAPI^+^/mCherry^−^) cells was compared between iNSPC monocultures and iNSPCs cocultured with mCherry^+^ MGs/MΦs using 12 data points (4 areas/sample, 3 samples/marker), as described [13].

To investigate the effect of MGs/MΦs on iNSPC differentiation, neurospheres derived from green fluorescent protein (GFP)^+^ iNSPCs were cultured alone or in combination with mCherry^+^ MGs/MΦs. Briefly, approximately 2–5 GFP^+^ neurospheres/well were plated onto mCherry^+^ MGs/MΦs in poly-L-lysine-coated dishes and incubated in neurobasal medium (Thermo Fisher Scientific) supplemented with bFGF, B-27 supplement (Thermo Fisher Scientific), and 2% FBS as described [1]. As a control, GFP^+^ neurospheres were plated onto mCherry^+^ ACs, ECs, or PCs, which were incubated under the same conditions. After fixation, the cocultures were immunostained with antibodies against GFP (1:1000, rabbit; Abcam) and mCherry (1:1000, chicken; Abcam). The areas of GFP^+^ neurites were evaluated per cell type in total 3 wells.

To further investigate the effect of MGs/MΦs on iNSPC differentiation, neurospheres derived from iNSPCs were cultured alone or in combination with mCherry^+^ MGs/MΦs. Briefly, approximately 5–10 neurospheres/well were plated onto mCherry^+^ MGs/MΦs and incubated in a neurobasal medium with bFGF, B-27 supplement, and 2% FBS. After fixation, the cocultures were immunostained with antibodies against Tuj1 (1:2000, rabbit; Abcam), microtubule-associated protein 2 (MAP2; 1:500, rabbit; Millipore), myelin basic protein (MBP; 1:100, mouse; R & D Systems, Minneapolis, MN, USA), and glial fibrillary acidic protein (GFAP; 1:500, rabbit; Abcam). The population of neurospheres that produced neurons (Tuj1^+^ cells and MAP2^+^ cells), astrocytes (GFAP^+^ cells), and oligodendrocytes (MBP^+^ cells) to all neurospheres were evaluated per well (9 wells/marker, 3 wells/group, [n = 3]).

### 2.5. Clodronate Liposome Treatment

For in vitro MG/MΦ depletion, clodronate encapsulated in liposomes, i.e., CD (+) liposomes (1 mM; Hygieia Bioscience, Osaka, Japan), was added to the culture medium. Liposomes without clodronate, i.e., CD (−) liposomes, were used as control.

### 2.6. Flow Cytometry

Analysis of MGs/MΦs with fluorescence-activated cell sorting (FACS) was performed using PE-conjugated antibodies against CD11b, CD86, and CD206, all obtained from Thermo Fisher Scientific with BD LSRFortessa™X-20 (BD Pharmingen), as described [6]. In some experiments, MGs/MΦs were treated with 10 ng/mL interleukin 4 (IL4; R & D Systems) and CD206^+^ MGs/MΦs were collected by FACS. Additionally, GFP^+^ iNSPCs collected by FACS were processed for microarray analysis.

### 2.7. Microarray Analysis

Control GFP^+^ iNSPC monocultures and GFP^+^ iNSPCs cocultured with MGs/MΦs were collected by FACS. Next, total RNA isolated from cells with the RNeasy Micro Kit (Qiagen, Hilden, Germany) was used in microarray analysis, as described [2,6,13]. The microarray data were analyzed using the Affymetrix Transcriptome Analysis Console [2,6,13] and Metascape gene ontology (GO) tool [14], as described. Pathway analysis was performed using WikiPathways, as described [6,15].

### 2.8. Statistical Analysis

Data were presented as means ± standard deviation. Comparisons among three or more groups were performed using a one-way analysis of variance, followed by Bonferroni post hoc tests. Comparisons between the two groups were performed using Student’s t-test. In all analyses, a *p* value of <0.05 was considered statistically significant.

## 3. Results

### 3.1. Distribution of iNSPCs and MGs/MΦs within and around Ischemic Regions following Stroke

**Figure 1:**
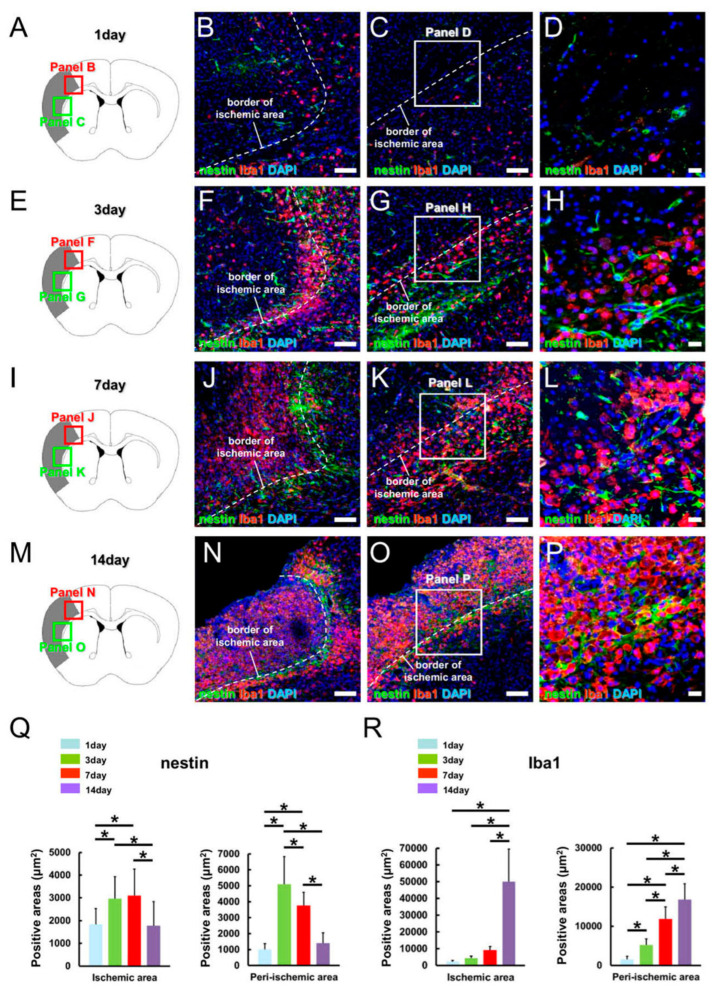
(**A**–**R**) Expression patterns (**A**–**P**) and semi-quantitative analysis (**Q**,**R**) of nestin^+^ iNSPCs and Iba-1^+^ MGs/MΦs after ischemic stroke in a mouse model. Double immunohistochemical staining for nestin (**B**–**D**,**F**–**H**,**J**–**L**,**N**–**P**) and Iba-1 (**B**–**D**,**F**–**H**,**J**–**L**,**N**–**P**) on poststroke days 1 (**A**–**D**), 3 (**E**–**H**), 7 (**I**–**L**), and 14 (**M**–**P**). Scale bars: 100 µm (**B**,**C**,**F**,**G**,**J**,**K**,**N**,**O**) and 20 µm (**D**,**H**,**L**,**P**). * *p* < 0.05 among groups (1, 3, 7, and 14 days after MCAO). Q and R, 27 data points (3 areas/section, 3 sections/brain, n = 3 mice for each day). Abbreviations: DAPI, 4′,6-diamidino-2-phenylindole; Iba-1, ionized calcium-binding adapter molecule 1; iNSPC, injury/ischemia-induced neural stem/progenitor cell; MCAO, middle cerebral artery occlusion; MG/MΦ, microglial cell/macrophage.

We first investigated the expression patterns of iNSPCs following an ischemic stroke in CB-17 mice, which develop highly reproducible ischemic regions restricted to the ipsilateral side of the cortex following MCAO [1,2,3,10]. Immunohistochemical analysis to determine the expression patterns of cells expressing the NSPC marker nestin within and around the ischemic region 1, 3, 7, and 14 days after stroke revealed that nestin^+^ cells were present in both the ischemic and peri-ischemic regions on poststroke day 1 (Figure 1A–D,Q). The size of nestin^+^ areas was significantly larger within and around the ischemic region on poststroke days 3 (Figure 1E–H,Q) and 7 (Figure 1I–L,Q) compared to that observed on poststroke day 1. However, the area of nestin^+^ cells was significantly smaller within and around the ischemic region on poststroke day 14 (Figure 1M–P,Q) compared to that observed on poststroke day 3 or 7.

We next investigated the expression patterns of Iba-1^+^ MGs/MΦs within and around the ischemic region on poststroke days 1, 3, 7, and 14. Immunohistochemical analysis revealed that a small population of Iba-1^+^ cells was present in both the ischemic and the peri-ischemic regions on poststroke 1 day (Figure 1A–D,R). The area of Iba-1^+^ cells in the peri-ischemic area gradually increased on poststroke days 3 (Figure 1E–H,R), 7 (Figure 1I–L,R), and 14 (Figure 1M–P,R). Additionally, the area of Iba-1^+^ cells within the ischemic region significantly increased on poststroke day 14 (Figure 1M–P,R).

### 3.2. MGs/MΦs Inhibit iNSPC Proliferation

**Figure 2:**
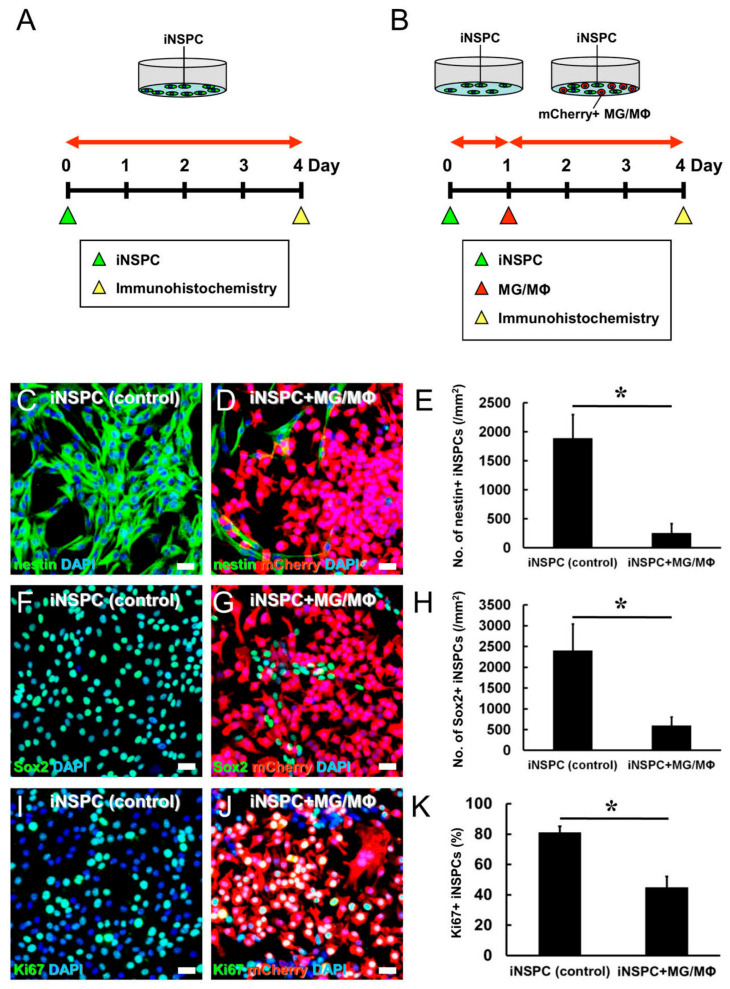
(**A**,**B**) Evaluation of iNSPC proliferation in monocultures (**A**) and cocultures with mCherry^+^ MGs/MΦs under direct cell–cell contact (**B**). (**C**–**E**) Immunostaining shows that the number of nestin^+^ iNSPCs (nestin^+^/mCherry^−^ cells) is significantly lower in the presence of mCherry^+^ MGs/MΦs (iNSPC + MG/MΦ group) compared to the iNSPC monoculture (control) (iNSPC group). Immunostaining for nestin (**C**,**D**), mCherry (**D**), and DAPI (**C**,**D**) is shown. (**F**–**H**) Immunostaining shows that the number of Sox2^+^ iNSPCs (Sox2^+^/mCherry^−^ cells) is significantly lower in the presence of mCherry^+^ MGs/MΦs (iNSPC + MGs/MΦs group) compared to the iNSPC monoculture (control) (iNSPC group). Immunostaining for Sox2 (**F**,**G**), mCherry (**G**), and DAPI (**F**,**G**) is shown. (**I**–**K**) Immunostaining shows that the ratio of iNSPC-derived proliferative cells (Ki67^+^/mCherry^−^ cells) to all iNSPCs (DAPI^+^/mCherry^−^ cells) is significantly lower in the presence of MGs/MΦs compared to the iNSPCs monoculture. Immunostaining for Ki67 (**I**,**J**), mCherry (**J**), and DAPI (**I**,**J**) is shown. Scale bar, 50 µm (**C**,**D**,**F**,**G**,**I**,**J**). * *p* < 0.05 compared to iNSPC monoculture (control); (**E**,**H**,**K**), n = 3 (12 data points) per group. Abbreviations: DAPI, 4′,6-diamidino-2-phenylindole; iNSPC, injury/ischemia-induced neural stem/progenitor cell; MG/MΦ, microglial cell/macrophage.

In our MCAO model, we observed an increase in the number of nestin^+^ cells within and around the ischemic region on poststroke days 3 and 7 days, which was followed by a decrease on poststroke day 14. Based on our observation of many Iba-1^+^ cells in these regions on poststroke day 14, we hypothesized that MGs/MΦs would negatively impact the fate of iNSPCs. To this end, iNSPCs were isolated from the ischemic region as described [11] and cultured alone (5 × 10^4^ cells/well) (Figure 2A) or in combination with mCherry^+^ MGs/MΦs added at an equal concentration (Figure 2B) in 6-well dishes. In coculture experiments, MGs/MΦs were added 24 h later after plating iNSPCs. Phase-contrast imaging conducted 1, 2, 3, and 4 days after incubation (Supplementary Figure S1) showed that not only iNSPCs (1 day, Supplementary Figure S1A,A’; 2 days, Supplementary Figure S1C,C’; 3 days, Supplementary Figure S1E,E’; 4 days, Supplementary Figure S1G,G’) but also MGs/MΦs, which were morphologically round in shape (1 day, Supplementary Figure S1B,B’; 2 days, Supplementary Figure S1D,D’; 3 days, Supplementary Figure S1F,F’; 4 days, Supplementary Figure S1H,H’), gradually increased over four days of coculture. The initial number of iNSPCs was not different between the iNSPC monocultures (Supplementary Figure S1A,A’) and the iNSPCs cocultured with MGs/MΦs (Supplementary Figure S1B,B’). However, on day 4 of incubation, the number of iNSPCs was decreased in cocultures containing MGs/MΦs (Supplementary Figure S1H,H’) compared to the iNSPC monocultures (Supplementary Figure S1G,G’).

To confirm this observation, the iNSPC monocultures (Figure 2A) and the iNSPCs cocultured with MGs/MΦs for 3 days (Figure 2B) were evaluated by immunostaining using antibodies against nestin, Sox2, and Ki67 (Figure 2C–K). The number of nestin^+^ iNSPCs (nestin^+^/mCherry^−^ cells) was significantly lower in cocultures containing MGs/MΦs (Figure 2D,E) compared to the iNSPC monocultures (Figure 2C,E). Similarly, the number of Sox2^+^ iNSPCs (Sox2^+^/mCherry^−^ cells) was significantly reduced by the presence of MGs/MΦs (Figure 2G,H) compared to the iNSPC monocultures (Figure 2F,H). Ki67^+^ proliferative cells were observed both in iNSPC monocultures and those cocultured with MGs/MΦs (Figure 2I,J). Additionally, Ki67 was observed in both mCherry^+^ and mCherry^−^ cells, indicating that Ki67 was expressed in MGs/MΦs as well as in iNSPCs. However, the number of iNSPC-derived proliferative cells (Ki67^+^/mCherry^−^ cells) was lower in iNSPCs cocultured with MGs/MΦs (Figure 2J) compared to the iNSPC monocultures (Figure 2I). The semi-quantitative analysis confirmed that the ratio of Ki67^+^ iNSPCs (Ki67^+^/mCherry^−^ cells) among all iNSPCs (DAPI^+^/mCherry^−^ cells) was significantly lower in iNSPCs cocultured with MGs/MΦs than in iNSPC monocultures (Figure 2K). These results indicated that MGs/MΦs regulated the number of iNSPCs in part by suppressing iNSPC proliferation.

We further examined whether direct cell–cell contact was essential for the observed impact of MGs/MΦs on iNSPCs by coculturing iNSPCs and MGs/MΦs under indirect cell–cell contact conditions using transwell inserts with 0.4 μm pore size, which would allow the passage of macromolecules but not cells. Briefly, one day after plating iNSPCs (3 × 10^4^ cells/well) in 12-well dishes, transwell inserts alone (Supplementary Figure S2A) or transwell inserts containing the same number of MGs/MΦs were placed in the dishes (Supplementary Figure S2B). Then, the iNSPCs alone (control) (Supplementary Figure S2A) or the iNSPCs co-cultured with MGs/MΦs were incubated for 3 days (Supplementary Figure S2B). Immunostaining revealed that, compared to the controls, the number of nestin^+^ iNSPCs was significantly, albeit very slightly, lower under indirect coculture conditions including MGs/MΦs (Supplementary Figure S2C). In light of the earlier findings in experiments evaluating direct cell–cell contact (Figure 2A–K), these results indicated that direct cell–cell contact rather than soluble factors was essential for the inhibitory effect of MGs/MΦs on iNSPC proliferation.

**Figure 3:**
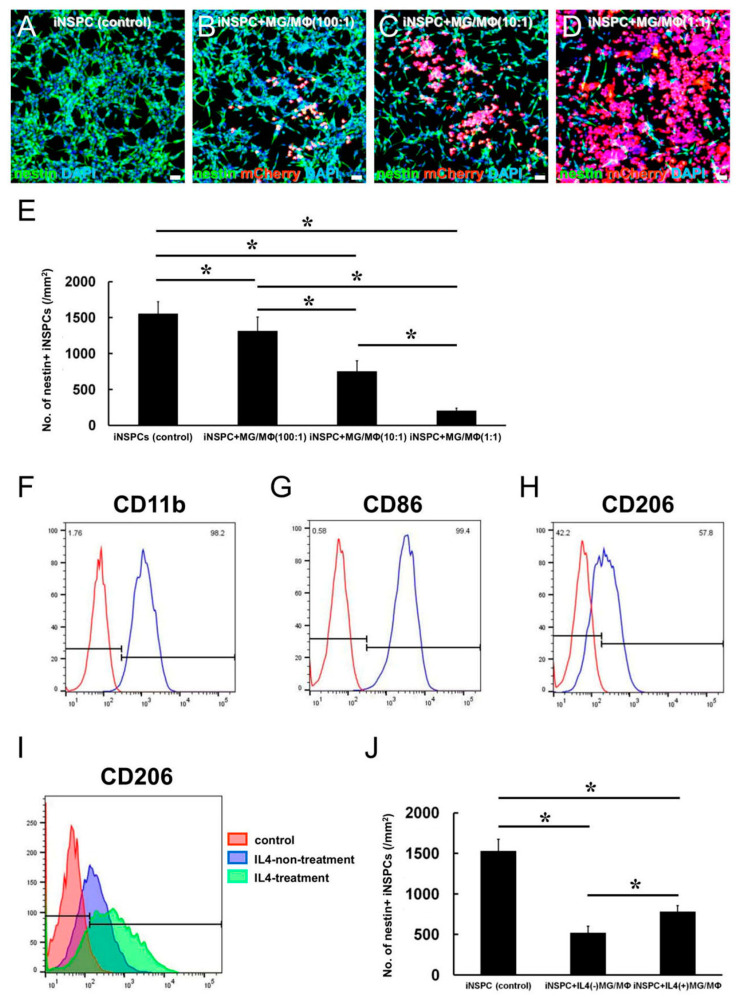
(**A**–**E**) Evaluation of iNSPC proliferation in monocultures (**A**,**E**) and cocultures with various doses of mCherry^+^ MGs/MΦs under direct cell–cell contact condition (**B**–**E**). Immunostaining shows that, compared to the monocultures (**A**), the number of nestin^+^ iNSPCs is significantly reduced in a dose-dependent manner in cultures containing higher numbers of mCherry^+^ MGs/MΦs: (**B**) 5 × 10^2^ MGs/MΦs, (**C**) 5 × 10^3^ MGs/MΦs, and (**D**) 5 × 10^4^ MGs/MΦs. Immunostaining for nestin (**A**–**D**), mCherry (**B**–**D**), and DAPI (**A**–**D**) is shown. (**F**–**J**) FACS analysis shows that the majority of MGs/MΦs express the conventional myeloid-lineage marker CD11b (98.2%) (**F**) and the M1 marker CD86 (99.4%) (**G**). The number of MGs/MΦs expressing the M2 marker CD206 (57.8%) (**H**) is increased following IL4 treatment (**I**). Compared to the iNSPC monoculture (control), the number of nestin^+^ iNSPCs is significantly decreased in cocultures with MGs/MΦs not treated with IL4, i.e., iNSPC + IL4(−) MGs/MΦs, and in those MGs/MΦs treated with IL4, i.e., iNSPC + IL4(+) MGs/MΦs (**J**). However, the number of nestin^+^ iNSPCs is significantly higher in iNSPC + IL4(+) MGs/MΦs than in iNSPC + IL4(−) MGs/MΦs (**J**). Scale bar, 50 µm (**A**–**D**). Control staining without primary antibody (red), positive cell population (blue) (**F**–**H**). * *p* < 0.05 among groups; (**E**,**J**), n = 3 (12 data points) per group (**E**), n = 4 (12 data points) per group (**J**). Abbreviations: DAPI, 4′,6-diamidino-2-phenylindole; FACS, fluorescence-activated cell sorting; iNSPC, injury/ischemia-induced neural stem/progenitor cell; MG/MΦ, microglial cell/macrophage.

To confirm this finding, iNSPCs (5 × 10^4^ cells/well) were cultured in 6-well dishes in the presence of various numbers of MGs/MΦs (5 × 10^2^, 5 × 10^3^, or 5 × 10^4^ cells/well) with direct cell–cell contact for 3 days. Compared to the control iNSPC monocultures (Figure 3A,E), the number of iNSPCs was significantly decreased in a dose-dependent manner in iNSPCs cocultured with increasing numbers of MGs/MΦs (Figure 3B–E).

MGs/MΦs can be roughly divided into M1 and M2 subtypes based on their predominant effects. M1-type MGs/MΦs exert cytotoxic effects, whereas M2-type MGs/MΦs exert cytoprotective effects [16]. Therefore, we next investigated the subtypes of MGs/MΦs. Consistent with our recent study [6], FACS analysis showed that almost all MGs/MΦs expressed the conventional myeloid-lineage marker CD11b (98.2%) (Figure 3F). Additionally, the vast majority of MGs/MΦs (99.4%) expressed the M1 marker CD86 (Figure 3G) whereas the rate of MGs/MΦs expressing the M2 marker CD206 was smaller (57.8%) (Figure 3H). These results suggested that most of the MGs/MΦs were likely cytotoxic rather than cytoprotective.

Thus, we investigated whether the negative impact of MGs/MΦs on iNSPCs was a result of a shift in MGs/MΦs toward the cytotoxic M1 type. To this end, MGs/MΦs were treated with IL4, which promotes a shift toward the M2 type [17]. FACS analysis showed that the rate of CD206^+^ MGs/MΦs increased from 57.3% to 79.4% following IL4 treatment (Figure 3I). Next, we sorted MGs/MΦs that were strongly positive for CD206 (CD206^++^ MGs/MΦs) following IL4 treatment, i.e., IL4(+) MGs/MΦs, and used CD206^+^ MGs/MΦs from cultures not treated with IL4, i.e., IL4(−) MGs/MΦs, as controls to coculture with iNSPCs. Although the number of nestin^+^ iNSPCs was significantly decreased in both coculture groups, the number of nestin^+^ iNSPCs was significantly higher in iNSPCs cocultured with IL4(+) MGs/MΦs than in those cocultured with IL4(−) MGs/MΦs (Figure 3J). These results suggested that the negative effect of MGs/MΦs on iNSPCs was attenuated following a shift of MGs/MΦs toward the M2 subtype.

### 3.3. MG/MΦ Depletion Inhibits the MG/MΦ-Mediated Negative Effects on iNSPC Proliferation

**Figure 4:**
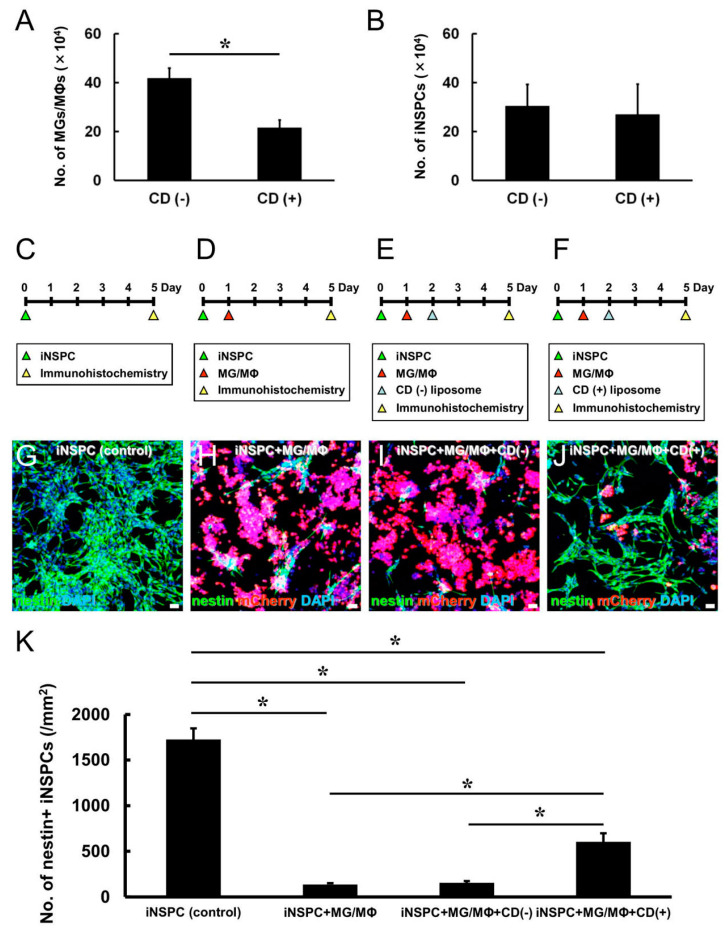
(**A**,**B**) The number of MGs/MΦs (**A**) and iNSPCs (**B**) after treatment with CD (−) or CD (+) liposomes. The number of MGs/MΦs is significantly lower in the CD (+) liposome group than in the CD (−) liposome group (**A**), whereas the number of iNSPCs is not significantly different between the CD (−) and CD (+) liposome groups (**B**). (**C**–**K**) iNSPC monocultures (**C**,**G**) and iNSPCs cocultured with mCherry^+^ MGs/MΦs (**D**–**F**,**H**–**J**) treated with CD (−) (**E**,**I**) or CD (+) liposomes (**F**,**J**). Immunostaining for nestin (**G**–**J**), mCherry (**H**–**J**), and DAPI (**G**–**J**) is shown. The number of nestin^+^ iNSPCs is significantly decreased in iNSPCs treated with MGs/MΦs compared to iNSPCs monocultures (**K**). However, the number of nestin^+^ iNSPCs after MG/MΦ treatment is significantly increased by CD (+) liposomes but not by CD (−) liposomes (**K**). Scale bar, 50 µm (**G**–**J**). * *p* < 0.05 compared to CD (−) (control); (**A**,**B**), n = 4 per group; * *p* < 0.05 among groups; (**K**), n = 3 (12 data points) per group. Abbreviations: CD, clodronate; DAPI, 4′,6-diamidino-2-phenylindole; injury/ischemia-induced neural stem/progenitor cell; MG/MΦ, microglial cell/macrophage.

Next, we determined whether MG/MΦ depletion by CD (+) liposomes could prevent the negative effect of MGs/MΦs on iNSPCs. First, 24 h after plating MGs/MΦs (3 × 10^4^ cells/well) in 24-well dishes, CD (+) or CD (−) liposomes (1 mM each) were added to the cultures and the number of MGs/MΦs was counted on post-treatment day 3. As shown in Figure 4A, the number of MGs/MΦs was significantly lower in cultures treated with CD (+) liposomes compared to the control cultures treated with CD (−) liposomes. Similarly, iNSPCs (3 × 10^4^ cells/well) were plated in 24-well dishes, followed by the addition of CD (+) or CD (−) liposomes (1 mM each) 24 h later. The evaluation of cultures on post-treatment day 3 revealed that the number of iNSPCs was not significantly different between the CD (−) and CD (+) liposome groups (Figure 4B). These results indicated that 1 mM of CD (+) liposomes was sufficient to suppress the proliferation of MGs/MΦs but not that of iNSPCs.

In the next experiment, iNSPCs (1.5 × 10^4^ cells/well in 24-well dishes, Figure 4C–F) which were cocultured in direct contact with MGs/MΦs (1.5 × 10^4^ cells/well, Figure 4D–F) for 24 h were further incubated with CD (−) and CD (+) or liposomes (Figure 4E,F, respectively) for 3 days. Compared to the iNSPC monocultures (Figure 4C,G,K), the number of nestin^+^ iNSPCs was significantly lower in iNSPCs cocultured with MGs/MΦs (Figure 4D,H,K). The number of nestin^+^ iNSPCs cocultured with MGs/MΦs was significantly increased by the addition of CD (+) liposomes (Figure 4F,J,K), which was not observed in cultures incubated with CD (−) liposomes (Figure 4E,I,K). These results showed that the suppressive effect of MGs/MΦs on iNSPC proliferation was inhibited by MG/MΦ depletion.

### 3.4. MGs/MΦs Specifically Inhibit the Differentiation of iNSPCs

**Figure 5:**
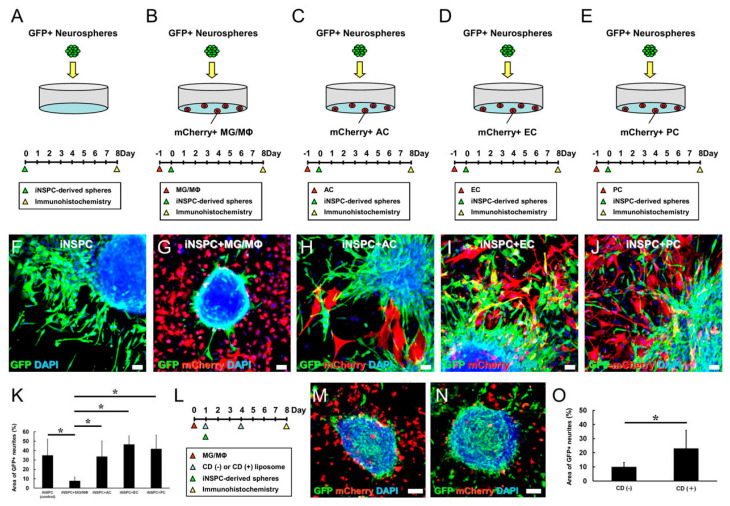
(**A**–**K**) Evaluation of differentiation potential of iNSPCs under direct cell–cell contact with specific cell types. GFP^+^ iNSPC-derived neurospheres incubated alone (**A**,**F**) or coincubated with mCherry^+^ MGs/MΦs (**B**,**G**), ACs (**C**,**H**), ECs (**D**,**I**), and PCs (**E**,**J**). Immunostaining for GFP (**F**–**J**), mCherry (**G**–**J**), and DAPI (**F**–**J**) is shown. (**K**) Immunostaining shows that, compared to the iNSPC-derived neurospheres alone (iNSPC, control), the GFP^+^ neurite area is significantly smaller in the presence of MGs/MΦs (iNSPC + MG/MΦ). (**L**–**O**) CD (−) or CD (+) liposomes were added to the medium of cultures containing iNSPC-derived neurospheres and MGs/MΦs. Immunostaining for GFP (**M**,**N**), mCherry (**M**,**N**), and DAPI (**M**,**N**) is shown. (**O**) Immunostaining shows that, compared to the cultures with CD (−) liposomes, the GFP^+^ neurite area is significantly larger in cultures treated with CD (+) liposomes. Scale bar, 50 µm (**F**–**J**,**M**,**N**). * *p* < 0.05 among groups; (**K**), n = 9 for iNSPC (control), n = 8 for iNSPC + MG/MΦ, n = 8 for iNSPC + AC, n = 15 for iNSPC + EC, n = 14 for iNSPC + PC; * *p* < 0.05 compared to CD (−) (control); (**O**), n = 10 for CD (−), n = 8 for CD (+). Abbreviations: AC, astrocyte; CD, clodronate; DAPI, 4′,6-diamidino-2-phenylindole; EC, endothelial cell; GFP, green fluorescent protein; iNSPC, injury/ischemia-induced neural stem/progenitor cell; MG/MΦ, microglial cell/macrophage; PC, pericyte.

Based on these results revealing that MGs/MΦs suppressed the proliferation of iNSPCs, we investigated whether the presence of MGs/MΦs impacted the differentiation of iNSPCs. GFP^+^ iNSPCs were incubated under conditions to promote the formation of neurosphere-like cell clusters. Next, these GFP^+^ neurospheres were plated in 24-well dishes and cultured either alone for 8 days (control cultures) (Figure 5A). In parallel, MGs/MΦs (1.0 × 10^4^ cells/well) were plated in 24-well dishes (Figure 5B), to which GFP^+^ neurospheres were added one day later; these cultures were incubated for 8 days (Figure 5B).

In control cultures containing only GFP^+^ neurospheres, the cells differentiated well and generated many neurites (Figure 5F). However, in cultures containing MGs/MΦs, the GFP^+^ neurospheres were poorly differentiated and neurite generation was rarely observed (Figure 5G). In support of these findings, the quantitative analysis confirmed that the GFP^+^ neurite area was significantly smaller in cultures containing MGs/MΦs compared to the control cultures (Figure 5K). These results indicated that MGs/MΦs suppressed the differentiation of iNSPCs.

We further investigated whether the observed negative effect on iNSPC differentiation was specific to MGs/MΦs or shared among other cell types in the brains. To this end, equal numbers (1.0 × 10^4^ cells/well) of mCherry^+^ ACs (Figure 5C), mCherry^+^ ECs (Figure 5D), and mCherry^+^ PCs (Figure 5E) were plated in 24-well dishes. One day later, GFP^+^ neurospheres were plated in the same dishes and incubated for eight days. The GFP^+^ neurospheres were well differentiated and produced many neurites in the presence of the evaluated cell types including ACs (Figure 5H), ECs (Figure 5I), and PCs (Figure 5J). The quantitative analysis revealed that the GFP^+^ neurite area was not significantly different in cultures containing ACs, ECs, or PCs compared to the control cultures containing only the GFP^+^ neurospheres (Figure 5K). These results indicated that the suppressive effect observed in iNSPC differentiation was specifically mediated by MGs/MΦs.

We next determined whether MG/MΦ-mediated suppression of iNSPC differentiation required direct cell–cell contact. GFP^+^ neurospheres were plated at the bottom of 12-well dishes. One day later, transwell inserts alone (Supplementary Figure S3A) or transwell inserts containing MGs/MΦs (2.0 × 10^4^ cells/well) were placed in the dishes (Supplementary Figure S3B). Then, the GFP^+^ neurospheres alone (control) (Supplementary Figure S3A) or the GFP^+^ neurospheres co-cultured with MGs/MΦs were incubated for 8 days (Supplementary Figure S3B). As shown in Supplementary Figure S3C, the GFP^+^ neurite area was not significantly different between the GFP^+^ neurospheres cultured alone and those incubated with MGs/MΦs, showing that direct cell–cell contact by MGs/MΦs, but not MG/MΦ-derived soluble factors, was essential for the suppressive effect of MGs/MΦs on iNSPC differentiation.

We further investigated whether the observed suppressive effect of MGs/MΦs on iNSPC differentiation was attenuated by CD (+) liposomes. MGs/MΦs (5.0 × 10^3^ cells/well) were plated in 24-well dishes. One day later, GFP^+^ neurospheres were added to the same dishes and incubated for seven days. Additionally, CD (−) or CD (+) liposomes were added to the cultures 1 and 4 days after the plating of MGs/MΦs (Figure 5L). Immunostaining (Figure 5M,N) and quantitative analysis (Figure 5O) showed that the GFP^+^ neurite area was significantly larger in cultures treated with CD (+) liposomes than in those treated with CD (−) liposomes, indicating that MG/MΦ depletion with CD (+) liposomes attenuated the negative effect of MGs/MΦs on iNSPC differentiation.

### 3.5. MGs/MΦs Inhibit Neural Differentiation, including Neurogenesis, in iNSPCs

**Figure 6:**
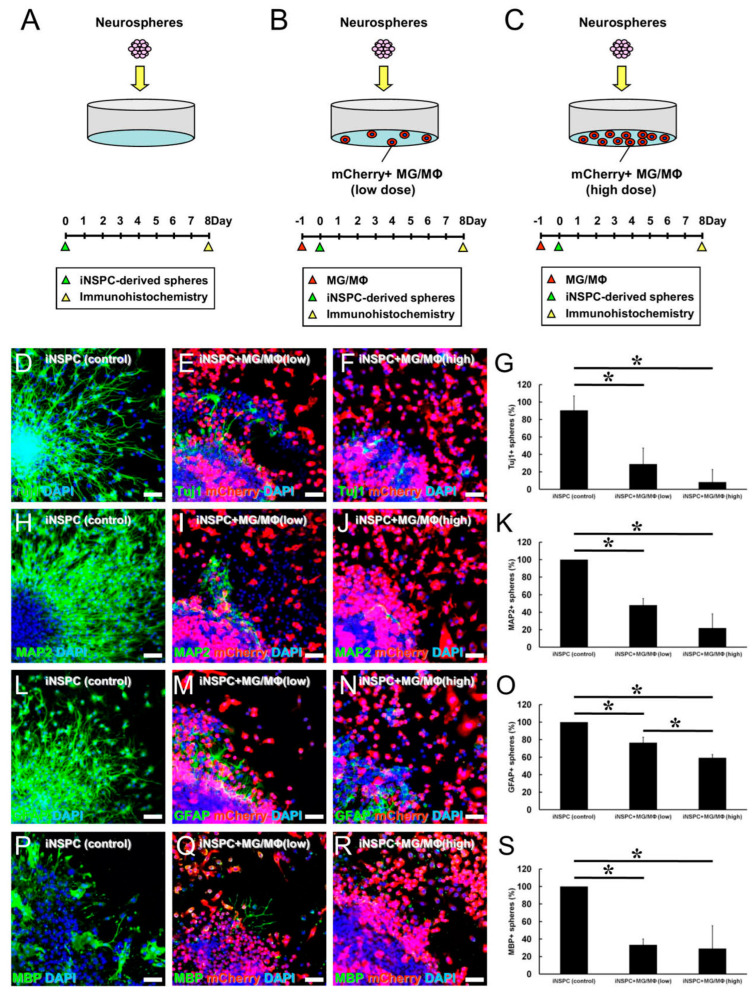
(**A**–**S**) Evaluation of neural differentiation potential of iNSPCs under direct cell–cell contact with low or high number of MGs/MΦs. iNSPC-derived neurospheres incubated alone (**A**,**D**,**H**,**L**,**P**) or with low (**B**,**E**,**I**,**M**,**Q**) or high number of mCherry^+^ MGs/MΦs (**C**,**F**,**J**,**N**,**R**). iNSPC-derived differentiated cells immunostained with Tuj1 (**D**–**G**), MAP2 (**H**–**K**), GFAP (**L**–**O**), and MBP (**P**–**S**). Immunostaining for Tuj1 (**D**–**F**), MAP2 (**H**–**J**), GFAP (**L**–**N**), MBP (**P**–**R**), mCherry (**E**,**F**,**I**,**J**,**M**,**N**,**Q**,**R**), and DAPI (**D**–**F**,**H**–**J**,**L**–**N**,**P**–**R**) is shown. Compared to the iNSPCs monoculture (control), the population of neurospheres producing Tuj1^+^ (**G**), MAP2^+^ (**K**), GFAP^+^ (**O**), and MBP^+^ cells (**S**) are significantly reduced by the presence of MGs/MΦs (iNSPC + MG/MΦ). Scale bar, 50 µm (**D**–**F**,**H**–**J**,**L**–**N**,**P**–**R**). * *p* < 0.05 among groups; (**G**,**K**,**O**,**S**), n = 3 per group. Abbreviations: DAPI, 4′,6-diamidino-2-phenylindole; GFAP, glial fibrillary acidic protein; GFP, green fluorescent protein; iNSPC, injury/ischemia-induced neural stem/progenitor cell; MAP2, microtubule-associated protein 2; MBP, myelin basic protein; MG/MΦ, microglial cell/macrophage.

Thus far, our data indicated that MGs/MΦs suppressed the differentiation of iNSPCs. However, it was unclear whether MGs/MΦs specifically inhibited the differentiation of neurons, astrocytes, or oligodendrocytes. To evaluate this effect, MGs/MΦs were plated in 24-well dishes at low (1.0 × 10^4^ cells/well, Figure 6B) and high (3.0 × 10^4^ cells/well, Figure 6C) numbers. One day later, neurospheres were added to the cultures, which were incubated for eight days. As a control, neurospheres were plated in 24-well dishes and incubated alone for eight days (Figure 6A).

The cultures were immunostained with antibodies against neuronal (Tuj1, MAP2), astrocytic (GFAP), and oligodendrocyte (MBP) markers, and the number of neurospheres with neurites that were positive for Tuj1, MAP2, GFAP, or MBP were counted. Compared with the control cultures, neuronal differentiation determined based on the population of neurospheres with Tuj1^+^ (Figure 6D–G) and MAP2^+^ cells (Figure 6H–K) was significantly inhibited by the presence of MGs/MΦs. Similarly, astrocytic differentiation assessed by the number of GFAP^+^ cells (Figure 6L–O) and oligodendrocytic differentiation assessed by the number of MBP^+^ cells (Figure 6P–S) were significantly inhibited by the presence of MGs/MΦs. Neuronal and oligodendrocytic differentiation was dramatically inhibited by the lower number of MGs/MΦs present in culture, and the ratios of Tuj1^+^, MAP2^+^, and MBP^+^ cells were not significantly different between the cultures incubated with the lower and higher numbers of MGs/MΦs (Figure 6G,K,S). The inhibitory impact of the lower number of MGs/MΦs on astrocytic differentiation was more limited compared to its inhibitory impact on neuronal and oligodendrocytic differentiation. The ratio of GFAP^+^ cells was significantly different between the cultures incubated with the lower and higher numbers of MGs/MΦs, and the ratio of GFAP^+^ cells was further decreased by the higher number of MGs/MΦs (Figure 6O). These findings indicated that, although MGs/MΦs suppressed both neuronal and glial differentiation of iNSPCs, a lower number of MGs/MΦs was sufficient to prevent the differentiation toward neuron and oligodendrocyte lineages, indicating that neuronal and oligodendrocytic differentiation was more easily inhibited by the presence of MGs/MΦs compared to astrocytic differentiation.

### 3.6. Gene Analysis Reveal That MGs/MΦs Negatively Regulate the Proliferation and Neuronal Differentiation of iNSPCs

**Figure 7:**
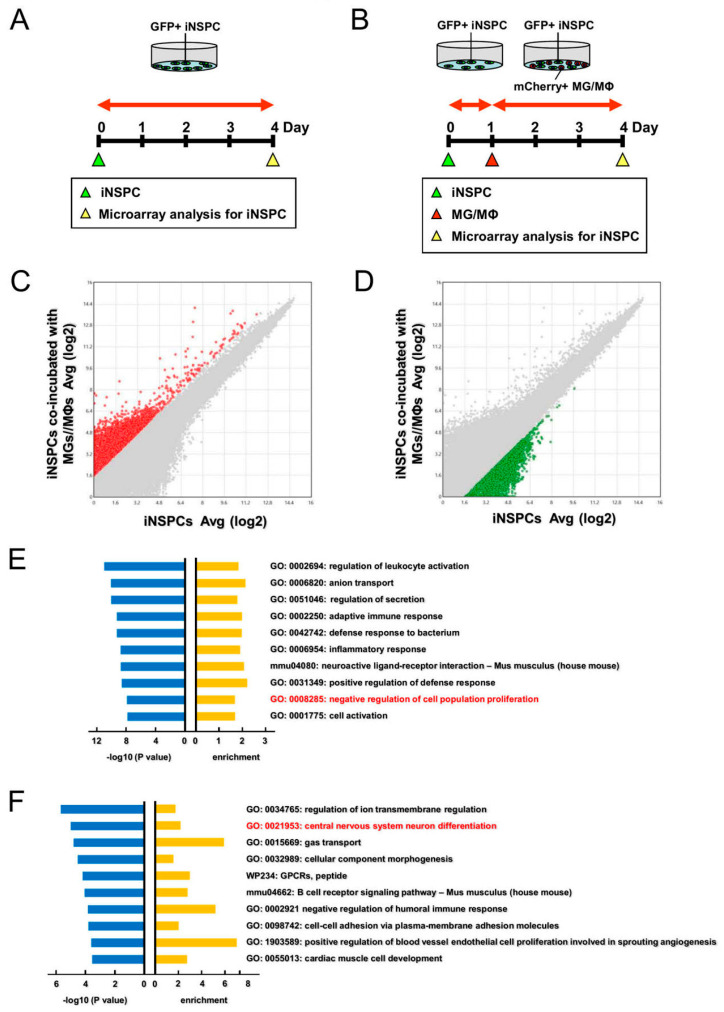
(**A**–**F**) Evaluation of changes in gene expression levels of iNSPCs after cell–cell contact with MGs/MΦs. GFP^+^ iNSPCs alone (**A**) or coculture with mCherry^+^ MGs/MΦs processed to sort for GFP^+^ cells by FACS for downstream microarray analysis (**B**). Scatter plot analysis shows the distribution of genes that are more than 3-fold higher (**C**, red plots) or more than 3-fold lower (**D**, green plots) in iNSPCs after coincubation with MGs/MΦs relative to iNSPCs alone. (**E**,**F**) The list of top 10 categories obtained by GO analysis of the genes of red (**E**) and green plots (**F**). Abbreviations: FACS, fluorescence-activated cell sorting; GFP, green fluorescent protein; GO, gene ontology; iNSPC, injury/ischemia-induced neural stem/progenitor cell; MG/MΦ, microglial cell/macrophage.

Finally, to investigate the mechanisms by which MGs/MΦs exerted the observed negative effects on iNSPCs in more detail, GFP^+^ iNSPCs monocultures (Figure 7A) and GFP^+^ iNSPCs cocultured with mCherry^+^ MGs/MΦs (Figure 7B) were incubated for 3 days. The GFP^+^ iNSPCs were then isolated by FACS for microarray analysis.

We first investigated the expression patterns of cell cycle-related genes. Pathway analysis showed that, although only 2 cell cycle-related genes (*Cdc25a* and *Mpeg1*) exhibited a significant upregulation of more than 2 fold in iNSPCs cocultured with MGs/MΦs compared to the iNSPCs monocultures, 10 cell cycle-related genes (*Ccna1*, *Ccnd3*, *Cdh1*, *Chek1*, *E2f2*, *Mcm6*, *Rb1*, *Rbl1*, *Tbc1d8*, and *Trp53*) exhibited a significant downregulation of more than 2 fold in iNSPCs cocultured with MGs/MΦs compared to the iNSPCs monocultures (Supplementary Figure S4). Supplementary Table S1 summarizes the fold changes in the expression levels of genes between the iNSPCs cocultured with MGs/MΦs and the iNSPCs monocultures.

We next investigated the expression patterns of apoptosis-related genes. Pathway analysis showed that, although 10 apoptosis-related genes (*Bcl2*, *Birc3*, *Fasl*, *Igf2*, *Ikbkb*, *Tnfrsf10b*, *Tradd*, *Traf1*, *Trp53*, and *Trp63*) were significantly downregulated for more than 2 fold in iNSPCs cocultured with MGs/MΦs compared to the iNSPCs monocultures, a total of 22 apoptosis-related genes (*Bid*, *Casp1*, *Casp4*, *Cflar*, *Diablo*, *Fas*, *Fasl*, *Gzmc*, *Igf1*, *Ikbkg*, *Irf1*, *Irf6*, Irf7, *Nfkbia*, *Nfkbib*, *Nfkbie*, *Pmaip1*, *Tnf*, *Tnfsf10*, *Traf1*, *Trp73*, and *Xiap*) were significantly upregulated for more than 2 fold in iNSPCs cocultured with MGs/MΦs compared to the iNSPCs monocultures (Supplementary Figure S5). Supplementary Table S2 summarizes the fold changes in the expression levels of these genes between the iNSPCs cocultured with MGs/MΦs and the iNSPCs monocultures. These results suggested that the presence of MGs/MΦs regulated cell proliferation and cell death in iNSPCs in part by modulating these genes.

Next, we performed GO analysis of the genes that were significantly higher in the iNSPCs cocultured with MGs/MΦs than in the iNSPC monocultures (>3 folds) (red dots, Figure 7C). Figure 7E shows the top 10 categories in order of fold changes, including “negative regulation of cell population proliferation” (GO: 0008285) (log10 p, −8.058; enrichment, 1.703). These results indicated that genes relevant to cell proliferation were downregulated in iNSPCs after their coculture with MGs/MΦs.

Similarly, we performed GO analysis of the genes that were significantly lower in the iNSPCs cocultured with MGs/MΦs than the iNSPC monocultures (<−3 folds) (green dots, Figure 7D). As shown in Figure 7F, the top 10 categories in order of fold changes included “central nervous system neuron differentiation” (GO: 0021953) (red font) (log10 p, −4.945; enrichment, 2.268), indicating the downregulation of genes related to neuronal differentiation in iNSPCs after coculture with MGs/MΦs. Taken together, these results suggested that MGs/MΦs suppressed cell proliferation and neuronal differentiation of iNSPCs in part by modulating specific genes belonging to these two categories.

## 4. Discussion

Ischemic stroke is a cerebrovascular disease that can cause severe neurological deficits due to cellular injury or cell death. Apoptotic cells release various cytokines and chemokines, which are involved in the activation of inflammatory cells such as MGs/MΦs that phagocyte debris and waste products including injured axons and myelin [18,19]. Previous reports showed that MGs/MΦs rapidly accumulated at the site of injury in response to inflammation within a couple of days after an ischemic stroke, with peak accumulation observed on poststroke days 4–14 [20,21,22]. Consistent with these reports, in the present study we observed abundant Iba-1^+^ MGs/MΦs within and around the ischemic area on poststroke days 7 and 14.

Only necrotic tissues and inflammatory cells are considered to be present within ischemic areas. However, using a mouse model of stroke, we previously demonstrated that, although mature neural cells including neurons, astrocytes, and oligodendrocytes underwent cell death after MCAO, endogenous iNSPCs, which can differentiate into these neural lineage cells, emerged within and around the ischemic area [2,3]. These findings indicate that not only inflammatory cells but also iNSPCs play an important role in the pathogenesis of an ischemic stroke.

Previous studies have shown that endogenous NSPCs in the subventricular zone (SVZ) migrate toward the site of injury following an ischemic stroke [23]. However, accumulating data demonstrate the limited migratory capacity of SVZ-derived NSPCs which fail to reach the site of injury after an ischemic stroke [24,25]. In support of this finding, we recently utilized genetic fate mapping analysis to show that most of the SVZ-derived NSPCs remained around the SVZ even after an ischemic stroke despite their migration toward the ischemic area [2]. However, iNSPCs could still be isolated from the ischemic area, indicating that these iNSPCs were likely derived from cells in situ but not from NSPCs in the SVZ. In fact, we and others demonstrated that iNSPCs originated in part from regionally derived reactive pericytes [3,10] and astrocytes [26,27].

Although the precise origins and traits of iNSPCs remain unclear, evidence that iNSPCs are present in ischemic areas indicates the potential of iNSPCs to regenerate damaged tissue following an injury such as an ischemic stroke. However, we previously demonstrated that iNSPCs at the site of injury gradually declined after an ischemic stroke [2,3]. Consistent with these results, in the present study we found that iNSPCs developing within and around the ischemic area declined within two weeks after MCAO, indicating that certain factors might negatively impact iNSPCs in this region during the post-ischemic period.

In support of this hypothesis, we previously demonstrated that certain subsets of CD4^+^ T lymphocytes were involved in the death of iNSPCs [28]. Under ischemic/hypoxic conditions, neurons are highly vulnerable and rapidly undergo cell death, which is also observed in mature astrocytes within ischemic areas [1]. However, a number of reactive ACs accumulate around ischemic areas [6]. Additionally, vascular lineage cells such as ECs and PCs are resistant to ischemia/hypoxia and survive within and around ischemic areas [1,6]. Thus, in pathological conditions such as postischemic stroke, it is possible that iNSPCs interact with ECs, PCs, ACs, and inflammatory cells such as MGs/MΦs. Therefore, in the present study, we investigated the roles of ECs, PCs, and ACs on iNSPCs. Contrary to our analyses revealing that the presence of MGs/MΦs negatively regulated the differentiation of iNSPCs, ECs, PCs, and ACs did not exert a negative impact on iNSPCs. These findings indicate that only MGs/MΦs among these cell types of the brain exerted a negative impact on iNSPCs.

Although the precise mechanism underlying the negative impact of MGs/MΦs on iNSPCs remains unclear, previous studies on embryonic and fetal brains during early developmental stages demonstrated that MGs/MΦs negatively regulated the number of NSPCs to inhibit excessive neurogenesis through several mechanisms including phagocytosis, inhibition of cell proliferation, and promotion of cell death [7,8,9]. Similarly, in the present study, we found alterations in the expression patterns of several genes related to cell death, cell proliferation, and neuronal differentiation in iNSPCs cocultured with MGs/MΦs. These results suggested that MGs/MΦs might modulate the iNSPC fate at least partially through the regulation of these genes after cell–cell interaction. Previous studies showed that proinflammatory cytokines, such as IL1β and IL18 released from MGs/MΦs, suppressed NSPC proliferation [29,30]. However, in the present study, the negative effect of MGs/MΦs on iNSPCs was prominently observed in conditions of direct cell–cell contact and not in conditions of indirect cell–cell contact. Although the cause of this discrepancy is unclear, several differences associated with coculture conditions, such as the duration of incubation, culture medium, and cell types of NSPCs and MGs/MΦs, might be contributors.

In the present study, MG/MΦ depletion by CD (+) liposomes attenuated the negative effect of MGs/MΦs on iNSPCs in vitro. However, whether MG/MΦ depletion is indeed beneficial for iNSPCs, such as the proliferation, survival, and neuronal differentiation of iNSPCs, requires further investigation in in vivo studies. Although the precise roles of MGs/MΦs in pathologic conditions are not fully elucidated, previous studies using animal models of stroke revealed that MG/MΦ depletion aggravated brain injury and inflammation after ischemia [31,32,33]. In contrast, studies using other animal models, such as intracerebral hemorrhage, showed that MG/MΦ depletion ameliorated brain injury and inflammation [34,35]. Therefore, whether MGs/MΦs exert harmful or beneficial roles in brain injury, including that which occurs in an ischemic stroke, remains a focus of debate [36].

Although the cause for this discrepancy between our findings and previous reports remains unclear, the use of different pharmacologic agents (e.g., CD (+) liposomes, PLX3397, and PLX5622) in different studies might be a contributor. For example, both PLX3397 and PLX5622 inhibit colony-stimulating factor-1 receptors, although PLX3397 also inhibits other receptors, such as c-kit [37]. Given that c-kit plays an important role in cellular homeostasis during repair after injury [38], inhibition of the c-kit signaling might have other consequences. Moreover, differences in various experimental conditions, such as the route of administration (e.g., oral versus intraperitoneal), duration (e.g., short-term versus long-term), and dose (e.g., low versus high dose) of pharmacologic agents, may account for the differences in the results of these studies. Alternatively, MG/MΦ response to pharmacologic agents might vary depending on the MG/MΦ phenotypes. MGs/MΦs can be generally categorized into M1 and M2 predominant states, which exhibit diverse traits: M1 MGs/MΦs exert proinflammatory effects, whereas M2 MGs/MΦs exert cytoprotective effects [39]. Indeed, in the present study we found that the negative effect of MGs/MΦs on iNSPCs was attenuated in iNSPCs cocultured with IL4-treated CD206^++^ MGs/MΦs. Although both M1 and M2 MGs/MΦs accumulate in the ischemic area, a previous study using mouse brains showed that the expression of M1 MGs/MΦs (CD16/32) peaked around poststroke day 14, while M2 MGs/MΦs (CD206) peaked around poststroke day 7 [22]. Thus, future studies should consider pharmacologic agents that selectively suppress M1 or M2 MGs/MΦs to elucidate the exact effect of MG/MΦ depletion on iNSPCs. However, accumulating studies have shown that MGs/MΦs have heterogeneous subpopulations [40] that do not fit either into M1 or M2 phenotypes [41]. Additionally, certain MGs/MΦs exhibit transitional (intermediate) phenotypes and simultaneously express M1 and M2 markers (e.g., CD86^+^/CD206^+^) [42,43], as observed in the current study. Moreover, previous studies revealed the presence of MGs/MΦs with transitional phenotypes (M1/M2 MGs/MΦs) under pathological conditions, including brain diseases [44,45]. Therefore, based on these findings, the precise roles of MGs/MΦs in relation to iNSPCs should be carefully investigated in further studies. 

The present study has several limitations. For example, we used MG/MΦ cell lines because of the difficulty of isolating primary cultured MGs/MΦs from mouse brains. However, it is possible that the features of MGs/MΦs differ between these cells. Thus, further studies are warranted to determine the precise roles of MGs/MΦs in relation to iNSPCs using primary cultured MGs/MΦs isolated from ischemic and non-ischemic regions.

In conclusion, we demonstrated that MGs/MΦs negatively regulated the proliferation and neuronal differentiation of iNSPCs isolated from the brains of mice after stoke induced by MCAO. Although the precise impact of MGs/MΦs on iNSPCs should be elucidated in future in vivo studies, the present study findings indicate that the regulation of MGs/MΦs should be considered as a therapeutic strategy to achieve iNSPC-based neural repair after an ischemic stroke.

## Data Availability

The data underlying this article will be shared upon reasonable request to the corresponding author.

## Supplementary Materials

The following supporting information can be downloaded at: https://www.mdpi.com/article/10.3390/cells12162040/s1.
