# Supplementary material for: Microglia Negatively Regulate the Proliferation and Neuronal Differentiation of Neural Stem/Progenitor Cells Isolated from Poststroke Mouse Brains
Source: Cells. 2023 Aug 10;12(16):2040. doi: 10.3390/cells12162040 (PMC10453473; doi:10.3390/cells12162040)
Supplement: Supplementary file 1 [file cells-12-02040-s001.zip › cells-2515549-supplementary.pdf]

Supplementary Figure S1

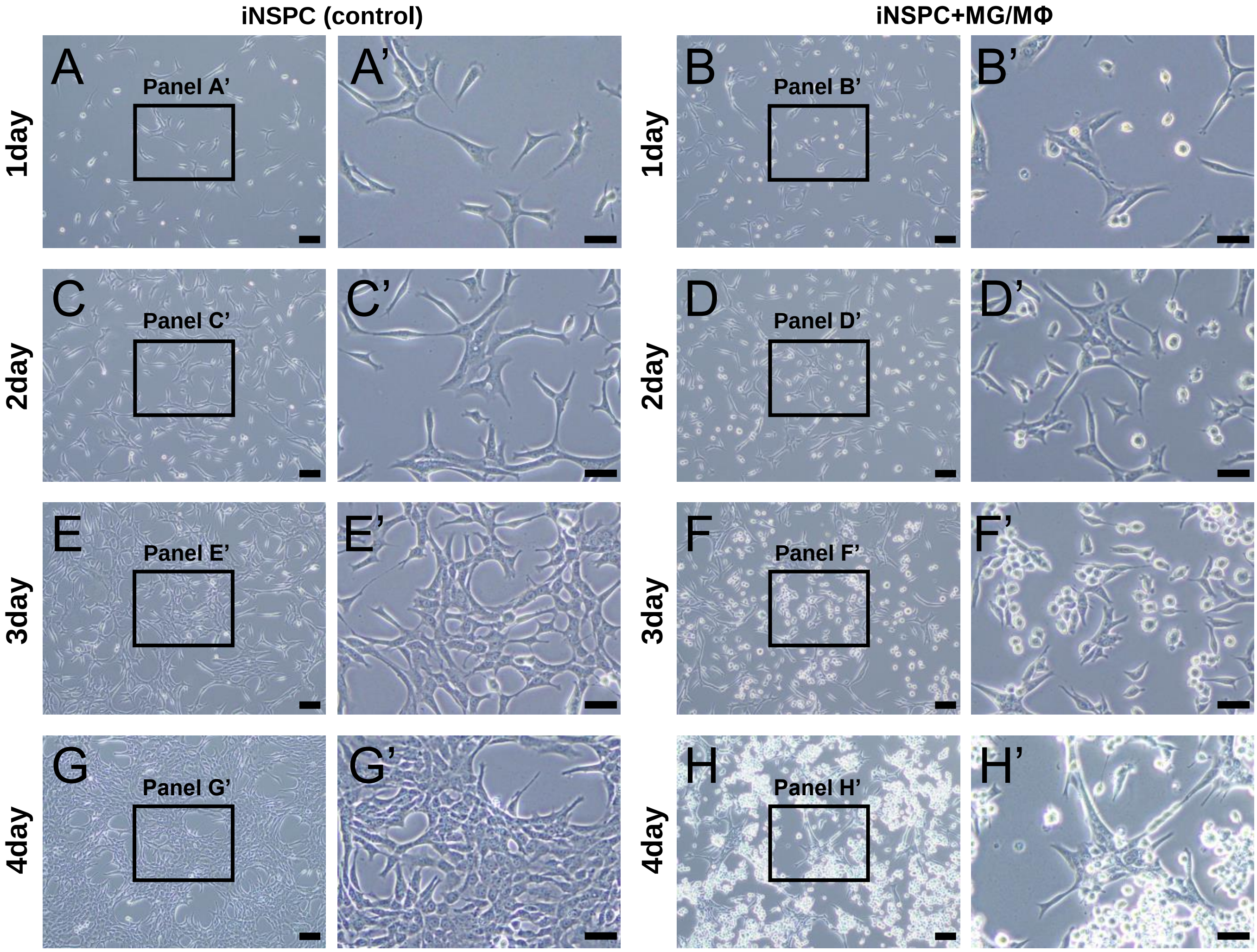

Supplementary Figure S2

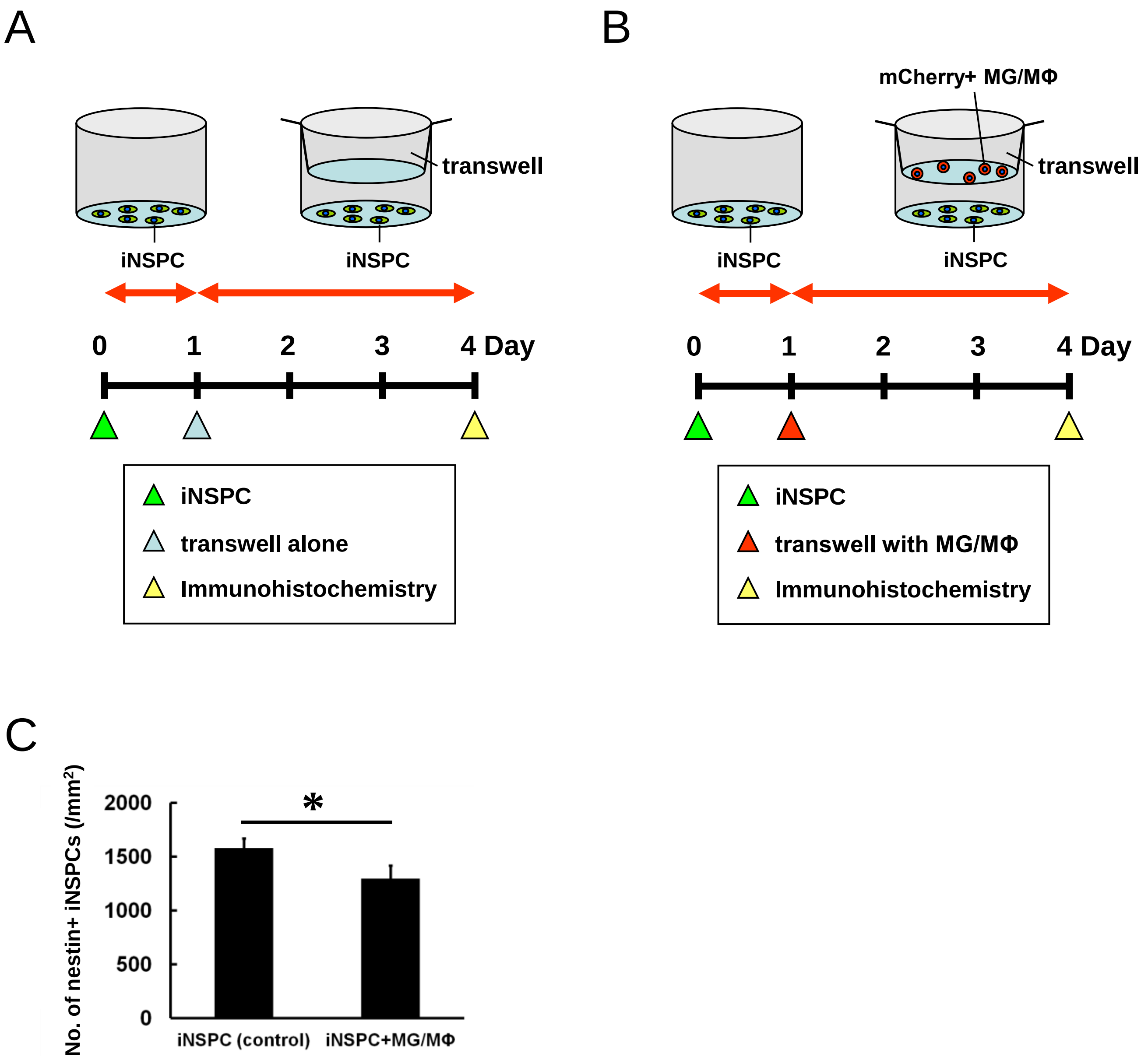

Supplementary Figure S3

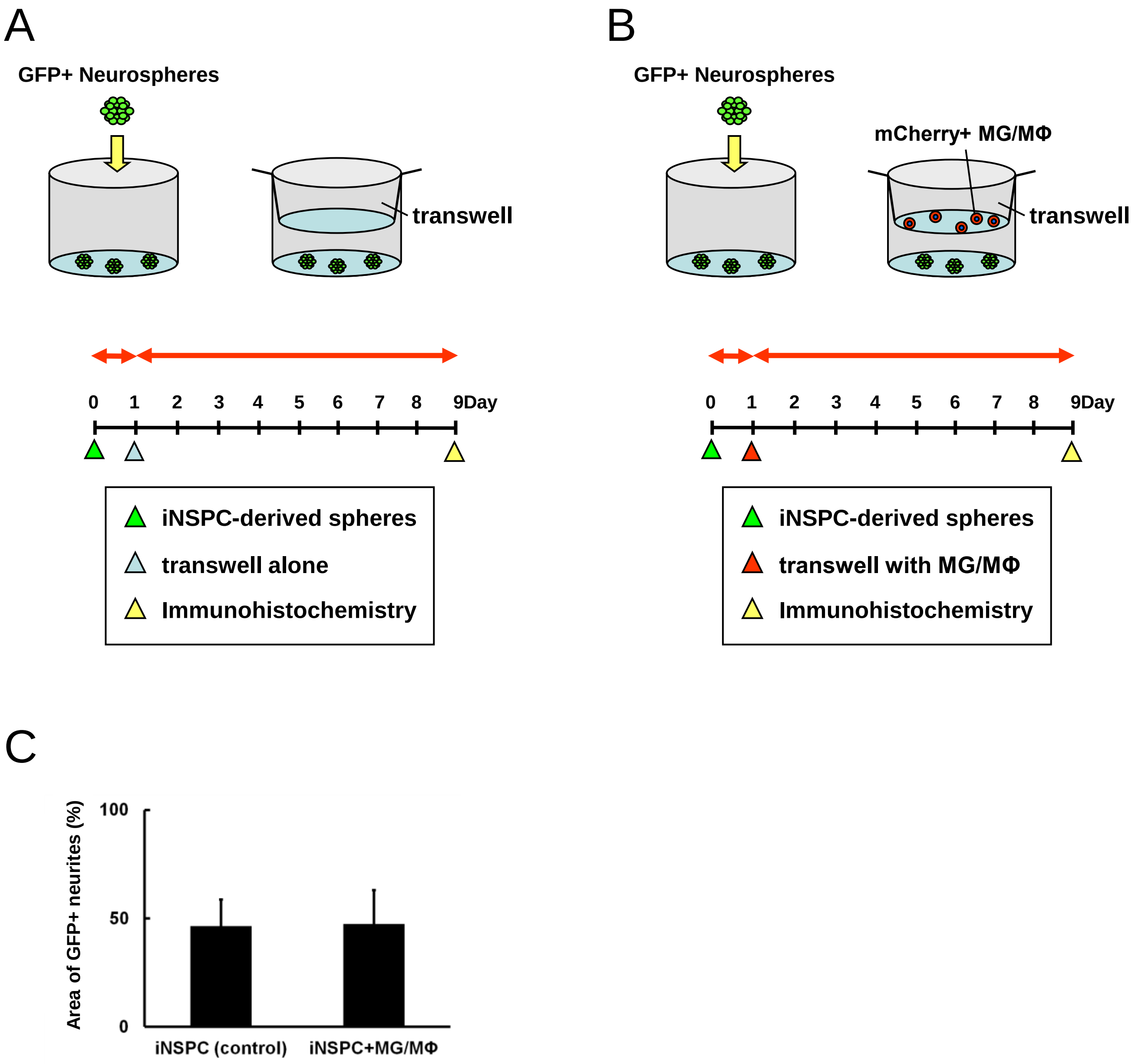

Supplementary Figure S4

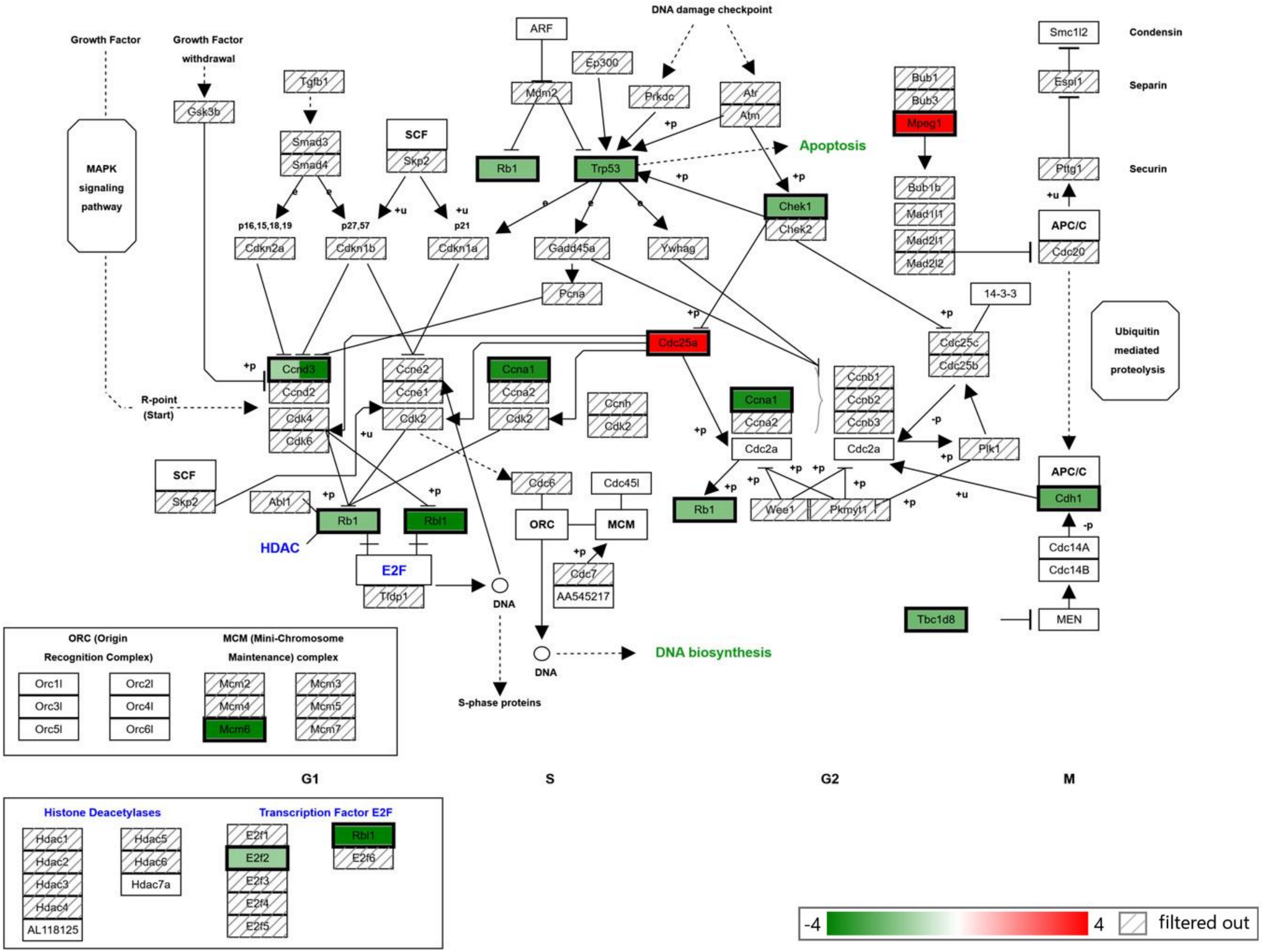

Supplementary Figure S5

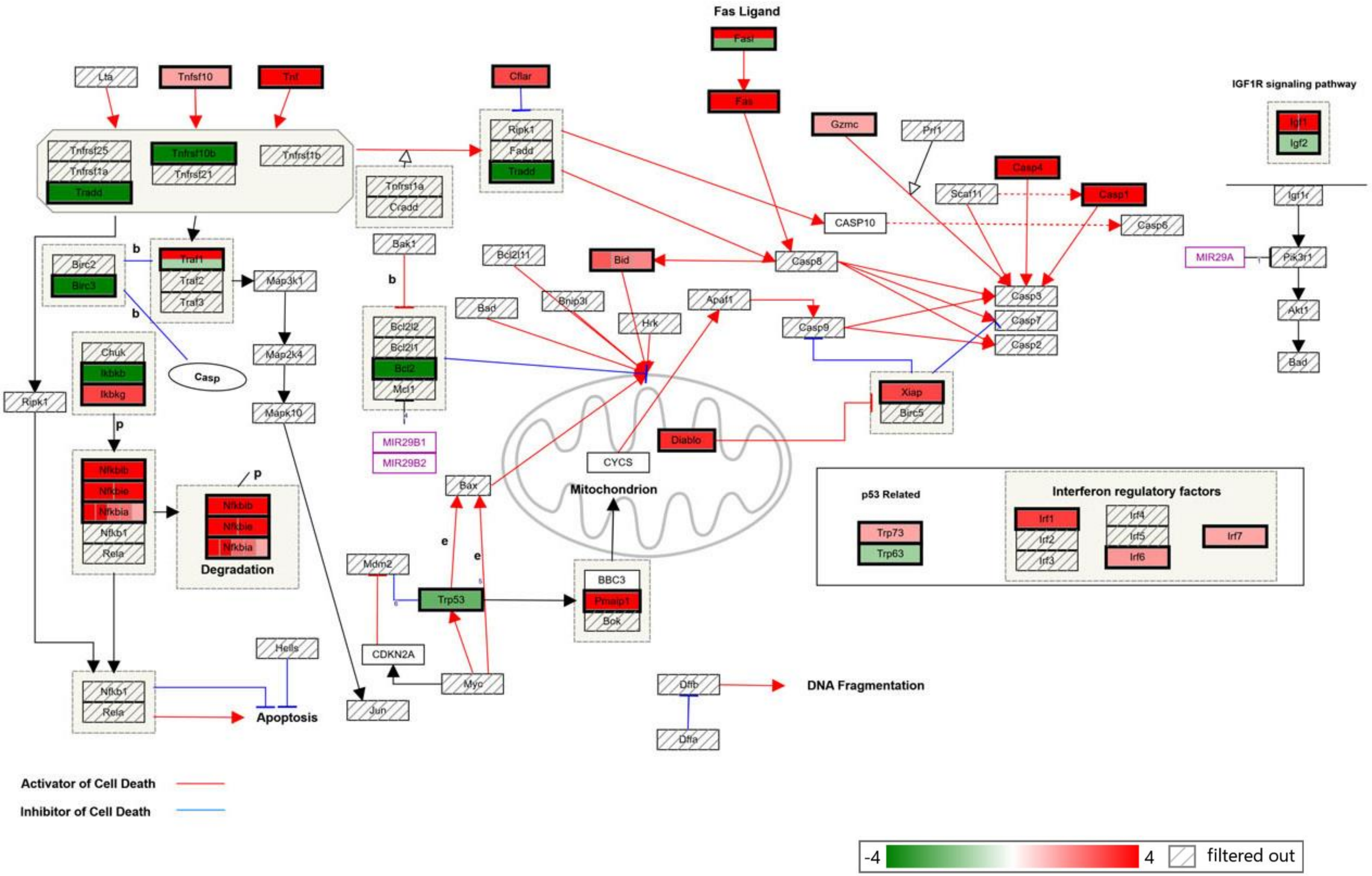

Supplementary Table S1

Comparison of cell cycle-related gene expression between iNSPCs co-incubated with MGs/MΦs and iNSPCs alone (iNSPCs with MGs/MΦs relative to iNSPCs alone)

Gene symbol	Gene name	ID	Fold change
Ccna1	cyclin A1	1449177_at	-3.7
Ccnd3	cyclin D3	1444323_at	-4.18
Cdc25a	cell division cycle 25A	1445995_at	4.87
Cdh1	cadherin 1	1448261_at	-2.89
Chek1	checkpoint kinase 1	1420032_at	-2.65
E2f2	E2F transcription factor 2	1436434_at	-2.16
Mcm6	minichromosome maintenance deficient 6 (MIS5 homolog, S. pombe) (S. cerevisiae)	1447756_x_at	-14.6
Mpeg1	macrophage expressed gene 1	1427076_at	20.77
Rb1	retinoblastoma 1	1444400_at	-2.45
Rbl1	retinoblastoma-like 1 (p107)	1454293_at	-4.18
Tbc1d8	TBC1 domain family, member 8	1455683_a_at	-2.65
Trp53	transformation related protein 53	1459781_x_at	-2.79

Supplementary Table S2

Comparison of cell cycle-related gene expression between iNSPCs co-incubated with MGs/MΦs and iNSPCs alone (iNSPCs with MGs/MΦs relative to iNSPCs alone)

Gene symbol	Gene name	ID	Fold change
Bcl2	B cell leukemia/lymphoma 2	1440770_at	-4.79
Bid	BH3 interacting domain death agonist	1417045_at	2.95
Bid	BH3 interacting domain death agonist	1448560_at	2.49
Bid	BH3 interacting domain death agonist	1447873_x_at	2.43
Birc3	baculoviral IAP repeat-containing 3	1425223_at	-5.05
Casp1	caspase 1	1449265_at	4.07
Casp4	caspase 4, apoptosis-related cysteine peptidase	1449591_at	5.75
Cflar	CASP8 and FADD-like apoptosis regulator	1453482_at	3.16
Diablo	diablo homolog (Drosophila)	1425768_at	3.51
Fas	Fas (TNF receptor superfamily member 6)	1460251_at	4.75
Fasl	Fas ligand (TNF superfamily, member 6)	1418803_a_at	4.66
Fasl	Fas ligand (TNF superfamily, member 6)	1449235_at	-2.66
Gzmc	granzyme C	1421256_at	2.02
Igf1	insulin-like growth factor 1	1419519_at	8.51
Igf1	insulin-like growth factor 1	1434413_at	7.01
Igf2	insulin-like growth factor 2	1448152_at	-2.12
Ikbkb	inhibitor of kappaB kinase beta	1445141_at	-9.33
Ikbkg	inhibitor of kappaB kinase gamma	1435647_at	3.2
Irf1	interferon regulatory factor 1	1448436_a_at	3.27
Irf6	interferon regulatory factor 6	1418301_at	2.23
Irf7	interferon regulatory factor 7	1417244_a_at	2.04
Nfkbia	nuclear factor of kappa light polypeptide gene enhancer in B cells inhibitor, alpha	1420088_at	2.73
Nfkbia	nuclear factor of kappa light polypeptide gene enhancer in B cells inhibitor, alpha	1420089_at	2.02
Nfkbia	nuclear factor of kappa light polypeptide gene enhancer in B cells inhibitor, alpha	1448306_at	4.01
Nfkbia	nuclear factor of kappa light polypeptide gene enhancer in B cells inhibitor, alpha	1449731_s_at	4.33

Nfkbia	nuclear factor of kappa light polypeptide gene enhancer in B cells inhibitor, alpha	1438157_s_at	2.79
Nfkbib	nuclear factor of kappa light polypeptide gene enhancer in B cells inhibitor, beta	1446718_at	8.73
Nfkbie	nuclear factor of kappa light polypeptide gene enhancer in B cells inhibitor, epsilon	1431843_a_at	3.93
Nfkbie	nuclear factor of kappa light polypeptide gene enhancer in B cells inhibitor, epsilon	1458299_s_at	4.37
Pmaip1	phorbol-12-myristate-13-acetate-induced protein 1	1418203_at	16.05
Tnf	tumor necrosis factor	1419607_at	4.85
Tnfrsf10b	tumor necrosis factor receptor superfamily, member 10b	1421296_at	-4.45
Tnfsf10	tumor necrosis factor (ligand) superfamily, member 10	1439680_at	2.09
Tradd	Tumor necrosis factor receptor type 1-associated DEATH domain protein; TNFRSF1A-associated via death domain	1439910_a_at	-6.2
Traf1	TNF receptor-associated factor 1	1423602_at	10.19
Traf1	TNF receptor-associated factor 1	1445452_at	-2.01
Trp53	transformation related protein 53	1459781_x_at	-2.79
Trp63	transformation related protein 63	1459581_at	-2.1
Trp73	transformation related protein 73	1427697_a_at	2.04
Xiap	X-linked inhibitor of apoptosis	1453385_at	3.19

Supplementary Figure legends

Figure S1

(A, C, E, G, A', C', E', G') Phase-contrast images showing the morphology of iNSPCs cultured alone and in combination with MGs/MΦs for 1 (A, A'), 2 (C, C'), 3 (E, E'), and 4 (G, G') days. (B, B', D, D', F, F', H, H') Phase-contrast images showing the morphology of iNSPCs cocultured with MGs/MΦs for 1 (B, B'), 2 (D, D'), 3 (F, F'), and 4 (H, H') days. Scale bar, 50 µm (A–H, A'–H'). Abbreviations: iNSPC, injury/ischemia-induced neural stem/progenitor cell; MG/MΦ, microglial cell/macrophage.

Figure S2

(A–C) Evaluation of iNSPC proliferation in monoculture (A) and coculture with mCherry$^+$ MG/MΦs under indirect cell–cell contact using transwell inserts (B). The number of nestin$^+$ iNSPCs (mCherry$^-$/nestin$^+$ cells) is significantly lower in the presence of MG/MΦs (iNSPC + MG/MΦs group) compared to the iNSPC monoculture (control) (C). *$p < 0.05$ compared to iNSPC (control); C, $n = 3$ (12 data points) per group. Abbreviations: iNSPC, injury/ischemia-induced neural stem/progenitor cell; MG/MΦ, microglial cell/macrophage.

Figure S3

(A–C) Evaluation of the differentiation potential of iNSPCs under indirect cell–cell contact using transwell inserts. GFP$^+$ iNSPC-derived neurospheres are incubated alone (control) (A) or coincubated with mCherry$^+$ MGs/MΦs (iNSPC + MG/MΦ) (B). The GFP$^+$ neurites area is not significantly different between the two groups. C, $n = 12$ for iNSPC (control), $n = 14$ for iNSPC + MG/MΦ. Abbreviations: GFP, green fluorescent protein; iNSPC, injury/ischemia-induced neural stem/progenitor cell; MG/MΦ, microglial cell/macrophage.

Figure S4

Pathway analysis of cell cycle-related genes in iNSPCs monocultures and iNSPCs cocultured with MGs/MΦs.

Figure S5

Pathway analysis of apoptosis-related genes in iNSPCs monocultures and iNSPCs cocultured with MGs/MΦs.

Supplementary Table S1

Comparison of cell cycle-related gene expression between iNSPCs with MGs/MΦs and iNSPCs alone (iNSPCs with MGs/MΦs relative to iNSPCs alone).

Supplementary Table S2

Comparison of apoptosis-related gene expression between iNSPCs with MGs/MΦs and iNSPCs alone (iNSPCs with MGs/MΦs relative to iNSPCs alone).
